# Longitudinal Development of Neuromuscular Performance and Multidirectional Speed in Youth Badminton Players: Evidence for Parallel Adaptation Trajectories

**DOI:** 10.3390/s26113393

**Published:** 2026-05-27

**Authors:** Mariola Gepfert, Artur Gołaś, Adam Maszczyk, Adam Zając, Anna Zwierzchowska

**Affiliations:** Institute of Sport Sciences, Academy of Physical Education, 40-065 Katowice, Poland; a.golas@awf.katowice.pl (A.G.); a.maszczyk@awf.katowice.pl (A.M.); a.zwierzchowska@awf.katowice.pl (A.Z.)

**Keywords:** racquet sports, reactive strength, motor abilities, sensor-based monitoring

## Abstract

This study examined long-term neuromuscular and multidirectional speed development in elite youth badminton players and evaluated whether developmental stage influences adaptation trajectories during systematic training. Thirty athletes were monitored over 16 months with repeated assessments at five time points and stratified into Younger (8–14 years) and Older (15–22 years) developmental groups. A comprehensive test battery assessed explosive strength, reactive strength, musculotendinous stiffness, and badminton-specific multidirectional speed. Data acquisition was performed using a multi-sensor approach, including force-platform-based jump analysis, accelerometry-based systems, and electronic timing gates, enabling the objective, high-resolution, and repeatable monitoring of neuromuscular performance. Significant time effects were observed across all sensor-derived performance variables (*p* < 0.001), indicating robust improvements in speed, power, and neuromuscular efficiency. Adaptation trajectories were predominantly linear, with no evidence of performance plateauing. Although older athletes maintained higher absolute performance levels, Time × Group interactions were largely absent, demonstrating parallel improvement rates across developmental stages rather than a catch-up effect in younger players. Linear mixed models confirmed equivalent improvement slopes despite baseline differences, and adjustment for body mass attenuated but did not eliminate age-group differences in jump performance. Exploratory analyses revealed substantial inter-individual variability, identifying responder phenotypes independent of age. These findings indicate that systematically progressed training supports sustained, linear neuromuscular adaptation across youth badminton development and highlight the importance of long-term, individualized monitoring over age-based expectations of accelerated responsiveness.

## 1. Introduction

Badminton is a high-intensity multidirectional sport that requires a complex interplay of speed, agility, explosive power, and precise coordination [1]. Success in this discipline depends on the simultaneous development of technical skills and a broad spectrum of motor abilities, the demands of which evolve dynamically throughout an athlete’s career [2]. Children and youth exhibit distinct neuromuscular characteristics compared to adults, often displaying higher variability in movement patterns and relying more on neural plasticity than on structural muscular adaptations [3]. While the general stages of long-term athlete development [4,5] are well documented, progressing from fundamental movement skills [6] in early childhood to sport-specific specialization in adolescence, specific data regarding the longitudinal susceptibility of young badminton players to training stimuli remain limited. Most existing research focuses on cross-sectional comparisons or short-term interventions [4,7,8,9,10], typically lasting between 8 and 12 weeks, leaving a gap in understanding how key motor abilities such as explosive strength, change-of-direction speed, and reactive agility develop over extended periods of time in elite youth populations. In particular, there is a scarcity of longitudinal studies incorporating multiple assessment time points that allow for a detailed description of developmental trajectories and the potential emergence of performance plateaus. Addressing this limitation, the present study employed a 16-month longitudinal design with five repeated evaluations, providing a more comprehensive perspective on neuromuscular development in elite youth badminton players. Recent advances in sensor-based technologies have enabled high-resolution and objective monitoring of neuromuscular performance in sport settings. Devices such as force platforms, wearable accelerometers, and electronic timing systems allow for the precise and repeatable data acquisition of key performance variables. Despite these technological developments, longitudinal studies integrating multi-sensor data across developmental stages in youth athletes remain limited.

Furthermore, the “catch-up” hypothesis, which suggests that younger athletes may exhibit accelerated gains owing to heightened neural adaptability [11], has not been rigorously tested in a controlled, long-term badminton setting [12]. In badminton, one of the most decisive offensive actions is the jump smash, which requires rapid vertical force production, efficient stretch-shortening cycle utilization, and coordinated whole-body kinetic chain transfer to maximize shuttle velocity and maintain post-landing recovery [13,14]. Although the present study did not directly assess smash velocity or stroke mechanics, the neuromuscular qualities evaluated here, such as countermovement jump performance, reactivity, musculotendinous stiffness, and multidirectional speed, are highly relevant to the physical capacities underpinning explosive take-off, landing control, and rapid repositioning during and after overhead attacking actions. Therefore, understanding the longitudinal development of these basic capacities may provide practically meaningful insight into the physical preparation strategies that support badminton-specific technical performance.

Agility and reactive strength are particularly critical in badminton, both during rally exchanges and in the execution and recovery phases of explosive overhead actions, such as the jump smash, where rallies are characterized by rapid accelerations, decelerations, and multiplanar lunges [15]. The stretch-shortening cycle (SSC) efficiency, manifested as reactive strength and leg stiffness, underlies these movements [16]. However, it is unclear whether the mechanisms of adaptation, such as the relationship between ground contact time and jump height, differ between preadolescent and adolescent players [17]. Recent findings indicate that while neuromuscular training effectively enhances performance in youth, the magnitude of SSC improvement may be maturation dependent [18,19,20]. Understanding these developmental trajectories is essential for optimizing training periodization in young athletes. If younger athletes respond differently to plyometric and speed stimuli than their older peers, training protocols should be differentiated not only by load but also by the qualitative nature of the stimulus [21]. Additionally, the heterogeneity of training responses, often categorized into “high” and “low” responders, adds another layer of complexity that requires investigation to move beyond one-size-fits-all coaching methodologies.

The primary objective of this study was to investigate longitudinal changes in neuromuscular performance in elite youth badminton players across two distinct developmental stages: pre-adolescence (8–14 years) and adolescence/young adulthood (15–22). Specifically, we attempted to determine whether athletes engaged in long-term systematic training exhibit parallel patterns of improvement in both groups or whether younger athletes demonstrate accelerated “catch-up” kinetics. The secondary aim was to explore potential mechanistic contributors to performance changes, particularly the role of contact time and musculotendinous stiffness, and to characterize inter-individual variability in adaptation patterns. We hypothesized that while both groups would improve significantly, the younger cohort would demonstrate greater relative gains in neuromuscular variables due to higher neural plasticity. From an applied perspective, these variables were selected because they represent basic physical qualities that may support critical badminton actions, including rapid court coverage and explosive overhead attacking movements such as the jump smash [13,14].

## 2. Materials and Methods

### 2.1. Participants

The longitudinal investigation was conducted over a 16-month period and involved youth badminton athletes stratified into two developmental cohorts: a younger group (YG) (n = 15; aged 8–14 years) and an older group (OG) (n = 15; aged 15–22 years), yielding a total sample size of N = 30. All participants competed at the national level and possessed substantial sport-specific training experience, defined as a minimum of two years for the younger cohort and seven years for the older one.

The study protocol was approved by the Research Ethics Committee for Scientific Research at the Academy of Physical Education in Katowice, Poland (approval number: 1-IV/2024), and was conducted in accordance with the Declaration of Helsinki (2013). All participants were fully informed about the study procedures, anticipated benefits, and potential risks prior to participation and provided written informed consent before the commencement of the investigation. For underage athletes, written consent was additionally obtained from parents or legal guardians. All collected data were anonymized and handled in strict confidence, with access restricted exclusively to the research team.

Participants were engaged in regular, structured badminton training as part of their high-performance development programs; however, the present study did not manipulate or control the training intervention, and therefore should be interpreted as an observational rather than an experimental study.

### 2.2. Study Design

The training exposure was embedded within the athletes’ regular, structured and periodized badminton training programs to ensure high ecological validity. Performance assessments were conducted at five standardized time points: baseline (T0) and four subsequent intervals spaced approximately four months apart (T1–T4). This design enabled a robust longitudinal evaluation of performance trajectories and inter-individual variability in developmental responses across successive phases of athletic maturation. All testing sessions were conducted under standardized indoor conditions and at similar times of day to minimize circadian and environmental variability.

### 2.3. Testing Protocol Standardization

To ensure high methodological rigor and minimize environmental confounders, all assessments were conducted at the same indoor testing facility using identical equipment and calibration procedures. Testing sessions were consistently scheduled at the same time of day to control for circadian variations in performance. Prior to data collection, participants completed a standardized warm-up protocol comprising 10 min of aerobic exercise, dynamic stretching, and submaximal jumps.

### 2.4. Measurement Procedures

A multi-sensor measurement approach was applied, integrating force-platform systems, wearable inertial sensors, and photoelectric timing systems to enable objective, high-resolution, and the repeatable data acquisition and monitoring of neuromuscular performance. A battery of standardized laboratory- and field-based tests was employed across all evaluation points to capture neuromuscular adaptations over time. Anthropometric characteristics were assessed to monitor growth-related changes across the study period. Body height was measured using a portable stadiometer to the nearest 0.1 cm, while body mass and body composition were evaluated using a multifrequency bioelectrical impedance analysis (InBody 720, InBody Co., Seoul, Republic of Korea). Measurements were conducted under standardized conditions, in the morning, with participants in a fasted state, in accordance with the manufacturer’s guidelines. Body mass values were subsequently included as covariates in the analysis of performance-related outcomes. Although body composition variables were recorded, only body mass was included in the final statistical models due to its relevance as a covariate and to avoid overparameterization of the analysis.

#### 2.4.1. Explosive Strength Assessment

All types of jumps were performed on the Chrono Jump platform (ChronoJump Boscosystem, Barcelona, Spain), a sensor-based force-platform system enabling precise and repeatable data acquisition of flight time and derived performance metrics, demonstrating excellent reliability (intra-class correlation coefficient of 0.999–1.000) [22]. All athletes performed three countermovement jumps (CMJs) and three single-leg jumps (SLJs) to evaluate bilateral and unilateral power output, respectively. A standardized rest interval of 30 s was applied between repetitions within each test, while a 3 min passive recovery period was enforced between different jump modalities to minimize the effects of fatigue accumulation. Testing procedures were conducted in accordance with previously established protocols [23]. Jump height derived from flight time was used as the primary indicator of explosive lower-limb power. Additionally, SLJ performance was used to assess unilateral power and potential asymmetries in force production. 

#### 2.4.2. Multidirectional Movement Assessment

To ensure uniform testing conditions, all speed assessments were conducted indoors in a standardized sports hall environment using electronic timing gates (Microgate Witty Gate photocells, Bolzano, Italy), which function as optical sensor systems enabling precise and repeatable measurement of movement time and high-resolution data acquisition. The gates were positioned at distances of 5, 10, and 20 m. Multidirectional movement speed was quantified through a series of badminton-specific sprint tasks designed to reflect the high-frequency accelerations, rapid directional changes, and court coverage demands characteristic of competitive match play.

The test battery included linear sprints over 5 and 10 m, lateral slide-step sprints over 5 m performed to both the right and left directions, crossover step sprints over 5 m executed bilaterally, as well as 10 m backward sprints. Each athlete completed two trials for each sprint condition, and the fastest time was retained for subsequent analysis. A standardized rest interval of 60–90 s was provided between trials, and 3 min of passive recovery was applied between different sprint conditions to minimize fatigue and ensure consistent performance [24].

#### 2.4.3. Neuromuscular Reactivity Assessment

Neuromuscular reactivity was assessed using the MyoTest system (MyoTest SA, Sion, Switzerland), a wearable inertial sensor utilizing accelerometry-based signal acquisition for objective and high-resolution monitoring of stretch-shortening cycle (SSC) performance. The sensor unit was securely positioned at the athlete’s waist during all jump trials to ensure accurate signal acquisition.

Participants performed standardized reactive jump tests consisting of repeated rebound jumps executed with minimal ground contact time and maximal vertical displacement. Each athlete completed two sets of three consecutive jumps, with the aim of maintaining reactive efficiency throughout the sequence.

Accelerometric data were recorded during each trial and processed using the manufacturer’s proprietary software (MyoTest PRO Software, version 1.0). The primary outcome variables included contact time (s), jump height (cm), lower-limb stiffness (as an estimate of musculotendinous stiffness during SSC actions), and reactivity (reflecting the efficiency of elastic energy utilization during repeated jumps).

For analysis, the optimal trial, defined as the sequence combining the highest jump height with the shortest contact time was retained. These variables were used to characterize SSC efficiency and rapid force production capacity relevant to badminton-specific movement demands.

### 2.5. Statistical Analysis

Statistical analyses were performed using SPSS software (version 28.0; IBM Corp., Armonk, NY, USA). Statistical significance was set a priori at α = 0.05 for all analyses.

To further examine the influence of anthropometric characteristics on performance outcomes, an analysis of covariance (ANCOVA) was performed with body mass included as a covariate. This approach allowed for the evaluation of group differences while statistically controlling for the potential confounding effect of body mass. Adjusted group means were compared, and effect sizes were interpreted accordingly.

#### 2.5.1. Data Screening and Assumptions

Prior to inferential analyses, data were screened for accuracy and distributional assumptions. Descriptive statistics are presented as means and standard deviations (M ± SD). Normality of distribution was assessed using the Shapiro–Wilk test, appropriate for small sample sizes (n < 50). Homogeneity of variances between groups was evaluated using Levene’s test, while homogeneity of variance–covariance matrices was examined using Box’s M test.

Potential multivariate outliers were identified using Mahalanobis distance (critical value = 16.42, df = 4), supported by univariate screening based on standardized residuals (z-scores) and Cook’s distance.

#### 2.5.2. Primary Longitudinal Analysis

To examine group-specific developmental trajectories across the 16-month period, a two-way mixed-design analysis of variance (ANOVA) with repeated measures was applied. Group (Younger vs. Older) served as the between-subject factor, while Time (T0, T1, T2, T3, T4) was specified as the within-subject factor.

The assumption of sphericity was assessed using Mauchly’s test. In cases where sphericity was violated, degrees of freedom were adjusted using Greenhouse–Geisser corrections. Significant main effects or interactions were followed by Bonferroni-adjusted post hoc pairwise comparisons. Effect sizes were reported as partial eta squared (ηp^2^) and interpreted as small (0.01), medium (0.06), or large (0.14) [25].

#### 2.5.3. Trajectory and Responder Analyses

To characterize the nature of longitudinal performance changes (e.g., linear progression or non-linear adaptation patterns), polynomial contrast analyses (linear and quadratic trends) were conducted. Inter-individual variability in training responsiveness was further explored using K-means cluster analysis. This unsupervised classification approach grouped athletes into distinct responder phenotypes (e.g., higher vs. lower responders) based on baseline performance levels and individual rates of change over time, complementing the mixed-model approach in examining adaptive heterogeneity independent of chronological age.

#### 2.5.4. Linear Mixed Models (LLM)

Linear Mixed Models (LMM) were employed to further examine longitudinal changes while accounting for the hierarchical structure of repeated measurements nested within individuals. This approach allows for the inclusion of both fixed effects (e.g., time, group) and random effects (participant-specific intercepts), thereby capturing inter-individual variability in baseline performance and response to training over time.

Compared to traditional repeated-measures ANOVA, LMM provides a more flexible framework for modeling within-subject dependencies, accommodates missing data without listwise deletion, and does not require the assumption of sphericity. This makes it particularly suitable for longitudinal sport science data, where individual adaptation trajectories may differ substantially.

The inclusion of random effects enabled the estimation of individual response patterns, supporting the interpretation of heterogeneity in training responsiveness across participants.

## 3. Results

Sensor-derived variables were analyzed to quantify longitudinal changes in neuro-muscular performance across the study period.

### 3.1. Participant Characteristics and Data Screening

The longitudinal investigation included N = 30 elite youth badminton athletes stratified into two developmental cohorts: Older (n = 15; 15–22 years; M = 18.3, SD = 2.1) and Younger (n = 15; 8–14 years; M = 11.6, SD = 1.9). All participants met the study-defined training experience criteria (≥7 years in the older cohort; ≥2 years in the younger cohort) and completed five standardized measurement time points over 16 months: T0 (baseline), T1 (4 months), T2 (8 months), T3 (12 months), and T4 (16 months). The protocol comprised 12 performance variables spanning speed/agility (n = 6), power (n = 3), and neuromuscular efficiency indices (n = 3).

Data were screened prior to inferential analyses. Assumptions for parametric analyses were met for 11 of 12 variables. Stiffness deviated from normality (Shapiro–Wilk *p* = 0.013) and was therefore additionally verified using non-parametric procedures for robustness. Homogeneity of variances was supported for all variables (Levene’s tests *p* > 0.05). Sphericity was acceptable for most outcomes; contact time and reactivity required Greenhouse–Geisser corrections. No multivariate outliers were detected (maximum Mahalanobis D^2^ = 8.56 < χ^2^ critical value 16.42, df = 4), and covariance matrix homogeneity was confirmed (Box’s M *p* = 0.642).

### 3.2. Baseline Developmental Differences (T0)

At baseline, the older cohort demonstrated clear performance advantages across nearly all domains. In speed and agility tasks, older athletes were faster in the linear 5 m sprint (1.127 ± 0.087 s vs. 1.296 ± 0.067 s; *p* < 0.001; d ≈ 2.2) and showed superior lateral and multidirectional court-movement performance (slide-step and cross-step tasks; all *p* < 0.001). Backward sprint performance also favored older athletes (1.387 ± 0.078 s vs. 1.613 ± 0.129 s; *p* < 0.001).

Power-related measures exhibited the largest developmental gaps. Older athletes displayed higher average rebound jump height (31.17 ± 3.74 cm vs. 27.73 ± 2.54 cm; *p* = 0.002; d ≈ 1.1) and substantially higher CMJ height (36.73 ± 4.92 cm vs. 26.60 ± 2.77 cm; *p* < 0.001). Unilateral jump performance was similarly higher in the older cohort (e.g., SLJ left: 18.39 ± 3.52 cm vs. 13.87 ± 2.12 cm). The reactivity index was also greater in older athletes (3.434 ± 0.521 vs. 2.951 ± 0.256; *p* = 0.003).

Notably, contact time during repeated jumping did not differ between cohorts at baseline (149.1 ± 25.7 ms vs. 151.7 ± 25.5 ms; *p* = 0.75), suggesting that the older group’s advantage was primarily driven by greater force production and jump output rather than shorter ground-contact duration.

### 3.3. Primary Longitudinal Effects of Time (T0–T4): Robust Improvements Across Domains

Repeated-measures ANOVA revealed strong main effects of Time across all 12 sensor-derived performance variables (all *p* < 0.001), with large effect sizes (η^2^ range: 0.513–0.709), indicating systematic improvements over the 16-month period across speed/agility, power, and neuromuscular efficiency outcomes. 

**Figure 1:**
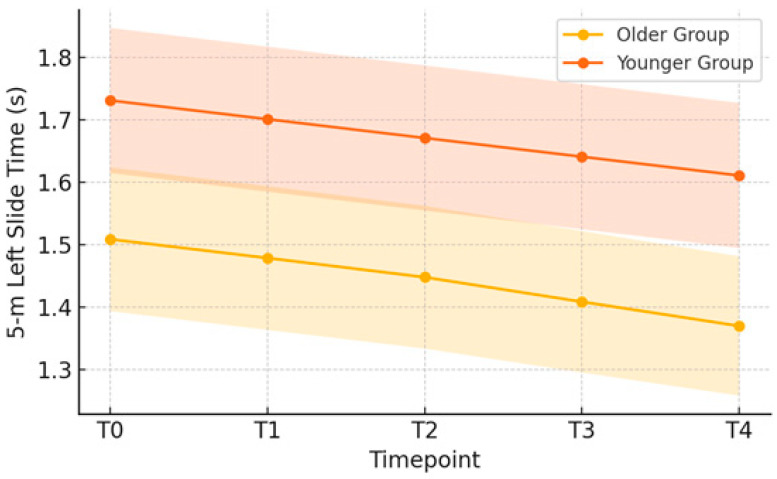
Changes in 5 m left slide-step speed over time for older vs. younger athletes. Both groups showed linear improvement (decreasing times) across the 16-month period, but the older group’s improvement was slightly greater, leading to a significant Time × Group interaction in this test. Error bands represent ±1 SD.

Speed and agility improved consistently. For the linear 5 m sprint, mean performance improved from 1.212 s at T0 to 1.091 s at T4 (~10.0% improvement; F(4,112) = 47.32, *p* < 0.001, η^2^ = 0.628). Comparable improvements were observed in lateral slide-step and cross-step tasks and backward sprinting, with typical gains in the ~7–9% range across the cohort (Figure 1).

Power and jump performance showed larger relative gains than speed. Average rebound jump height increased from 29.45 cm to 34.65 cm (+17.6%; F(4,112) = 52.13, *p* < 0.001, η^2^ = 0.651). CMJ height increased by 6.00 cm from T0 to T4 (+18.9%; F(4,112) = 59.87, *p* < 0.001, η^2^ = 0.682). Single-leg jump performance showed the largest proportional gain (mean +4.42 cm; +27.6%; F(4,112) = 68.34, *p* < 0.001, η^2^ = 0.709).

Neuromuscular efficiency indices improved in parallel with performance outcomes. Contact time decreased from 150.37 ms to 142.37 ms (−5.3%; F(4,112) = 44.21, *p* < 0.001, η^2^ = 0.612), while reactivity increased from 3.192 to 3.712(+16.3%; F(4,112) = 48.76, *p* < 0.001, η^2^ = 0.635). Stiffness also increased significantly over time (Kruskal–Wallis *p* < 0.001), supporting progressive musculotendinous adaptation.

### 3.4. Developmental Group Effects and Parallel Improvement Trajectories (Group and Time × Group)

Across the observation period, significant main effects of Group were observed for most outcomes (typical η^2^ ≈ 0.214–0.347; *p* < 0.01), indicating that older athletes maintained superior absolute performance levels across time in both speed/agility and power measures. Contact time remained the notable exception, with no meaningful group difference (*p* = 0.566; η^2^ = 0.012).

Crucially, Time × Group interactions were largely absent, indicating parallel improvement trajectories across developmental stages despite baseline performance gaps. Of the 12 measures, only Slide Step Right showed a statistically significant interaction (F(4,112) = 2.87, *p* = 0.026, η^2^ = 0.093), while Backward Speed demonstrated only a marginal trend (*p* = 0.081). All remaining outcomes showed non-significant interactions (*p* range: 0.107–0.887; η^2^ range: 0.008–0.065).

For prototypical outcomes (CMJ and linear 5 m sprint), interaction effects were negligible (CMJ: F(4,112) = 0.54, *p* = 0.706; linear 5 m sprint: *p* > 0.5). In practical terms, both groups improved by similar absolute magnitudes (e.g., CMJ +6.0 cm; linear 5 m sprint −0.12 s), supporting the interpretation of parallel developmental adaptation rather than a “catch-up” effect.

### 3.5. Trajectory Analysis: Sustained Linear Adaptation Without Plateau

Polynomial contrast analyses indicated a consistent pattern of strictly linear improvement across the entire 16-month period. Linear contrasts were highly significant for all outcomes (all *p* < 0.001), accounting for the majority of temporal variance (η^2^ range: 0.795–0.893). Quadratic components were uniformly non-significant (all *p* > 0.05), providing no evidence of acceleration, deceleration, or plateau effects. This pattern supports sustained performance progression across successive training phases.

### 3.6. Secondary and Exploratory Analyses: Confirmation, Covariate Adjustment, Responsiveness, and Mechanisms

#### 3.6.1. Linear Mixed Models (Confirmatory)

Linear mixed-model analyses confirmed the findings of the repeated-measures ANOVA. For linear 5 m sprint speed, the model estimated a younger-group baseline of 1.297 s (SE = 0.087) with a linear improvement rate of −0.030 s per 4-month interval (SE = 0.002). The older group demonstrated a significant baseline advantage (−0.169 s; SE = 0.081; *p* = 0.046), while the Time × Group interaction was non-significant (*p* = 0.731), indicating equivalent improvement rates across developmental stages.

For CMJ height, the younger-group baseline was estimated at 29.60 cm (SE = 2.34), with a consistent improvement slope of 1.50 cm per 4-month interval (SE = 0.089). Although the group effect was large (β = 10.13 cm; *p* = 0.003), the Time × Group interaction remained non-significant (*p* = 0.668). Random-effects estimates indicated substantial inter-individual variability in baseline CMJ performance, but minimal variability in individual improvement slopes, supporting the presence of parallel developmental trajectories.

#### 3.6.2. ANCOVA: Role of Body Mass in Developmental Differences

ANCOVA adjusting for body mass demonstrated that developmental (group) differences in jump outcomes persisted after covariate control (e.g., adjusted *p*-values: CMJ *p* = 0.007; SLJ left *p* = 0.005; average rebound jumps *p* = 0.009). Adjustment reduced group-effect magnitudes by approximately 24–32%, suggesting that body mass explained part—but not the majority—of age-related performance advantages. Body mass correlated with jump outcomes (standardized β ≈ 0.321–0.458), but did not meaningfully predict speed/agility outcomes (all *p* > 0.05), consistent with predominantly neuromuscular rather than mass-driven speed differences.

#### 3.6.3. Responsiveness: Responder Rates and Trajectory Phenotypes (Exploratory)

Responder classification suggested high training responsiveness across domains with minimal age dependence. The proportion of jump responders (≥1 SD improvement) was 90.0% (27/30), with comparable rates in older (86.7%) and younger athletes (93.3%; *p* = 0.412). Linear 5 m sprint responder rate was 80.0% (24/30) with identical proportions across cohorts. Multi-domain responsiveness (≥1 SD improvement in ≥2 domains) was observed in 73.3% (22/30), with no significant age-group difference (*p* = 0.267).

K-means clustering of CMJ trajectories identified three phenotypes: High responders (n = 8), Typical responders (n = 16), and Slow responders (n = 6), differing primarily in baseline CMJ and slope magnitude. Cluster membership was not significantly associated with age group (*p* = 0.343), supporting the interpretation that individual responsiveness was largely independent of chronological age.

#### 3.6.4. Mechanistic Analysis: Contact Time as a Partial Mediator of CMJ Improvements (Exploratory)

Mediation analysis indicated that improvements in CMJ height over time were partially mediated by reductions in contact time. Training duration positively predicted CMJ height (β = 1.500 cm per 4-month interval; *p* < 0.001) and negatively predicted contact time (β = −2.0 ms per 4-month interval; *p* < 0.001). Contact time was inversely associated with CMJ height (β = −0.345 cm per ms; *p* < 0.001). The indirect (mediated) effect via contact time was β = 0.690 cm per 4-month interval, accounting for 31.5% of the total time effect, while a substantial direct effect remained, indicating the presence of additional mechanisms beyond contact time improvements.

### 3.7. Effect Sizes and Training Responsiveness

Within-group effect sizes from T0 to T4 revealed large to very large training effects across most performance domains. Speed and agility measures showed large improvements (Hedges’ g ≈ −0.81 to −1.76), while power-related variables demonstrated particularly strong gains, especially in the younger cohort (CMJ: g ≈ 2.07; single-leg jump: g ≈ 1.99). In contrast, contact time exhibited only small effects (g ≈ −0.31), indicating constrained plasticity of ground-contact duration. Overall, mean within-group effect sizes were greater in younger athletes (g = 1.78) than in older athletes (g = 1.19; *p* = 0.036).

Between-group effect sizes remained stable or increased slightly over time. For CMJ, between-group effects increased by ~8% from T0 to T4, while sprint-speed differences increased by ~24%, indicating that although both groups improved substantially, baseline developmental advantages were maintained.

### 3.8. Exploratory Correlational and Responder Analyses

Exploratory correlation analyses revealed clear biomechanical and neuromuscular coupling across performance domains. Body mass demonstrated significant positive associations with jump-related outcomes, including CMJ height (r = 0.458, *p* < 0.001) and single-leg jump performance (r = 0.382, *p* < 0.001), whereas associations with speed-related variables were negligible or non-significant (all *p* > 0.05) (Table 1). This pattern indicates that sprint and agility performance were primarily determined by neuromuscular factors rather than body mass per se.

Strong within-task correlations were observed for paired movement patterns, including linear and backward sprint performance (r = 0.734, *p* < 0.001), right–left slide-step performance (r = 0.687, *p* < 0.001), and right–left cross-step performance (r = 0.654, *p* < 0.001). These findings suggest domain-general mechanisms underlying multidirectional agility adaptations rather than direction-specific learning effects (Table 1).

Responder analysis indicated high training responsiveness across domains with minimal age dependence. The proportion of responders (≥1 SD improvement from baseline) was 90.0% for jump performance and 80.0% for linear sprint speed, with no significant differences between younger and older cohorts. Multi-domain responsiveness (≥1 SD improvement in at least two performance domains) was observed in 73.3% of participants, and more than half of the sample (53.3%) demonstrated improvements across all three key domains (CMJ, sprint speed, and reactivity) (Table 2). Athletes classified as non-responders or partial responders exhibited higher baseline performance values, consistent with regression-to-the-mean effects rather than absence of training adaptation.

K-means clustering of CMJ growth trajectories identified three distinct responsiveness phenotypes: high responders (26.7%), typical responders (53.3%), and slow responders (20.0%), differing primarily in baseline CMJ values and rates of improvement. Importantly, cluster membership was not associated with developmental group (*p* = 0.343), indicating that individual variability in training responsiveness was largely independent of chronological age and likely influenced by person-specific factors such as baseline neuromuscular status, training history, and recovery capacity (Table 2).

### 3.9. Exploratory Mediation Analysis: Contact Time as a Mechanistic Pathway

Exploratory mediation analysis was conducted to examine mechanistic pathways underlying training-induced improvements in CMJ height (Table 3). Training duration exerted a strong total effect on CMJ performance (β = 1.50 cm per 4-month interval, *p* < 0.001). Training duration was also associated with a significant reduction in ground contact time, which in turn was inversely related to CMJ height (both *p* < 0.001) (Table 3).

The indirect effect mediated through contact time accounted for 31.5% of the total training effect on CMJ, while the remaining 68.5% represented direct effects independent of contact time efficiency (Table 3). These findings indicate that although improvements in stretch-shortening cycle efficiency contribute meaningfully to CMJ enhancement, the majority of training-related gains are attributable to additional neuromuscular mechanisms, such as increased force production capacity, enhanced rate of force development, improved neuromuscular coordination, and structural adaptations of the muscle–tendon unit.

## 4. Discussion

The present study provides a rare long-term perspective on neuromuscular and multidirectional speed development in elite youth badminton players, based on repeated assessments spanning 16 months. The principal finding is that robust, predominantly linear improvements in explosive strength, reactive strength, and sport-specific movement speed were observed over the course of long-term exposure to systematic training and development across both pre-adolescent and adolescent athletes. These performance qualities closely align with the physiological and biomechanical demands of competitive badminton, which involve frequent accelerations and decelerations, rapid changes in direction, and repeated jump- and lunge-based actions performed under time pressure and limited recovery [7,26,27,28,29]. The use of integrated sensor technologies enabled precise and high-resolution tracking of neuromuscular adaptations over time, highlighting the value of multi-sensor monitoring in long-term athlete development.

A key observation was the absence of performance plateauing across the observation period. Linear trends accounted for the majority of variance in all outcome measures, indicating that appropriately managed progression of training load and task complexity can sustain meaningful neuromuscular adaptation across successive training phases in youth racket sports. This finding challenges the notion that gains in speed and power necessarily attenuate over time during adolescence and emphasizes the importance of long-term planning rather than short-term intervention effects.

### 4.1. Developmental Trajectories: Parallel Improvement Rather than Catch-Up

A main objective of the study was to determine whether younger athletes would exhibit accelerated rates of improvement relative to their older counterparts, potentially narrowing baseline performance gaps through heightened neural plasticity or lower training age. Contrary to a “catch-up” hypothesis, the results supported a parallel trajectory model: rates of improvement observed over time were statistically comparable between age groups for the majority of variables, while older athletes retained a stable absolute performance advantage. The use of Linear Mixed Models further strengthens the interpretation of these findings by explicitly accounting for inter-individual variability in training response. By modeling individual trajectories rather than relying solely on group means, this approach enables a more nuanced understanding of adaptation patterns, including the presence of both high and low responders within each age group. In this context, the lack of a significant group × time interaction suggests that training responsiveness is not strictly determined by chronological age, but rather reflects individual variability in neuromuscular adaptation.

This pattern is consistent with contemporary long-term athlete development models, which emphasize that strength, power, speed, and agility remain trainable throughout childhood and adolescence when training stimuli are scaled to the athlete’s developmental readiness and integrated with sports practice [30]. It also aligns with meta-analytic evidence showing that resistance and plyometric training elicit meaningful improvements in youth athletes across a wide age range, with maturation influencing the magnitude of performance but not the capacity to adapt [12,19,21].

The persistence of between-group differences likely reflects the combined influence of biological maturation and accumulated sport-specific experience. Maturation is accompanied by increases in muscle strength and progressive stiffening of the muscle–tendon complex, enabling greater absolute force and power output [31,32]. Concurrently, greater exposure to badminton-specific movement patterns may enhance coordination and movement economy in tests resembling court footwork demands [8,33]. The limited number of group-by-time interactions observed most notably in one lateral slide-step condition likely reflects direction-specific technical or laterality-related constraints rather than generalized age-dependent sensitivity to training. However, it should be acknowledged that the present study did not include direct markers of biological maturation, such as Peak Height Velocity (PHV). Therefore, the observed developmental patterns likely reflect a combined influence of structured training and natural growth-related processes, which cannot be fully disentangled within the current design.

### 4.2. Neuromuscular Adaptations: Stiffness, Reactivity, and Force Expression

Improvements in reactive strength and musculotendinous stiffness emerged as consistent mechanistic features across both age groups, suggesting enhanced utilization of the SSC. In badminton, SSC-dominant actions such as the split step, rapid landing–rebound sequences, and repeated lunge recoveries are fundamental to effective court coverage and shot preparation [7,29,34]. Training-induced increases in tendon stiffness may improve force transmission and elastic energy reutilization, supporting more rapid force expression during short ground-contact actions [35].

From a developmental perspective, the observation of stiffness increases in younger athletes is particularly noteworthy. Although childhood is typically characterized by relatively compliant tendons, both mechanical loading and maturation influence tendon properties across adolescence. Importantly, disproportionate adaptation between muscle and tendon has been proposed as a risk factor for overuse injury if training loads progress too rapidly [36]. In the present cohort, long-term improvements in SSC-related measures occurred without reported injury events, supporting the feasibility of sustained plyometric and agility training when progression is gradual and appropriately supervised [1,35].

The mediation analysis further refined interpretation of jump performance adaptations. Reductions in contact time accounted for approximately one-third of the training-related improvement in CMJ height, while the majority of gains were attributable to mechanisms independent of contact-time efficiency. These adaptive changes be partially explained by training-induced increases in musculotendinous stiffness, which enhance force transmission and elastic energy reutilization during the stretch-shortening cycle. In this context, improved stiffness may facilitate more effective storage and return of elastic energy, thereby contributing to greater jump height without necessarily reducing ground contact time.

Additionally, long-term neuromuscular adaptations such as increased maximal force production, improved rate of force development, and enhanced intermuscular coordination likely play a central role in this effect. These mechanisms may allow athletes to generate higher impulse within sport-relevant time constraints, supporting improvements in explosive performance that are not solely dependent on contact-time reductions. This pattern is consistent with the view that long-term power development is driven primarily by enhanced force production capacity and rate of force development within sport-relevant time constraints [7,37], whereas reductions in contact time may become progressively smaller once fundamental movement competency is established [38]. In badminton-specific contexts, greater force-producing capacity may translate into faster repositioning, improved braking control, and enhanced stability during high-intensity rally exchanges [39,40].

### 4.3. Integration of Speed, Power, and Multidirectional Movement

The observed coupling between speed, power, and change-of-direction measures suggests that neuromuscular qualities developed in an integrated manner rather than as isolated attributes. Agility in sport encompasses rapid whole-body movement involving changes in velocity or direction in response to task demands and therefore reflects the combined influence of strength, power, technique, and coordination [41,42]. In badminton, effective change-of-direction performance depends on braking and re-acceleration capacity within highly constrained spatial environments and asymmetric footwork patterns [8,43]. Previous work in youth badminton players has similarly reported meaningful associations between sprint, jump, and on-court change-of-direction performance, supporting the use of combined neuromuscular testing batteries for long-term monitoring [8,44,45]. From a practical badminton perspective, these findings may also be relevant to the physical determinants of jump-smash execution. The jump smash is not only a technical stroke but also a high-power movement task requiring explosive lower-limb impulse, rapid force transmission through the kinetic chain, and efficient landing-to-recovery mechanics. In this context, the observed improvements in CMJ performance, reactive strength, musculotendinous stiffness, and multidirectional speed may reflect enhanced physical readiness to support more effective take-off, stroke preparation, and post-smash court repositioning. However, because smash mechanics and shuttle velocity were not measured directly, these interpretations should be considered functionally informed rather than confirmatory.

### 4.4. Inter-Individual Variability and Responder Phenotypes

Despite strong group-level improvements, substantial inter-individual variability was evident, with cluster analysis identifying distinct responsiveness phenotypes that were not explained by chronological age. Such heterogeneity in training response is well documented and reflects interactions among genetic predisposition, baseline performance, recovery capacity, and training history [2,46]. Methodological considerations are also important, as responder classifications may be influenced by measurement error and normal within-person variability, underscoring the value of repeated longitudinal assessment [47,48].

Nevertheless, the high proportion of responders across domains highlights the practical relevance of individualized monitoring. Evidence from youth training interventions suggests that tailoring training stimuli—through adjustments in volume, intensity, exercise selection, or recovery emphasis—may help optimize adaptation in slower responders while maintaining a coherent long-term development pathway [49]. This approach is consistent with integrative neuromuscular training models that seek to enhance performance while managing injury risk throughout youth sport participation [50,51].

It should also be acknowledged that responder classifications derived from clustering approaches may not be temporally stable across extended developmental periods. In youth athletes, responsiveness to training is likely influenced by dynamic factors such as biological maturation, fluctuations in training load, recovery status, and transitional phases in development. As a result, an athlete classified as a higher or lower responder at one stage may exhibit different adaptation patterns over time. Future research should therefore consider time-resolved or longitudinal classification approaches to better capture potential shifts in responder status and to enhance the effectiveness of individualized monitoring strategies.

### 4.5. Practical Implications and Limitations

Collectively, these findings support the inclusion of year-round, progressively periodized strength, plyometric, and multidirectional speed training within high-performance badminton development from pre-adolescence through adolescence. For coaches, these findings suggest that monitoring jump-based and SSC-related variables may be useful not only for general athletic development but also for supporting explosive overhead actions such as the jump smash, particularly when integrated with technical stroke training and badminton-specific footwork drills.

Monitoring SSC-related indices such as reactive measures, contact time, and stiffness proxies may assist coaches in interpreting performance changes mechanistically and guiding load progression, particularly during periods of rapid growth when muscle–tendon balance may be challenged [52]. Given the directional constraints and laterality demands of badminton, practitioners should also consider unilateral and multi-planar braking–re-acceleration drills and periodically assess left–right asymmetries using sport-specific tests [42,53].

Several limitations warrant consideration. An additional limitation is that no direct measure of badminton-specific technical performance, such as jump-smash velocity, shuttle speed, or stroke kinematics, was included; therefore, the practical transfer of the observed neuromuscular adaptations to overhead attacking performance should be interpreted indirectly. The sample size, although typical for elite longitudinal research, limited statistical power to detect small interaction effects. The absence of a non-training control group precludes complete separation of training effects from maturation changes, and future studies should incorporate direct biological maturation markers, such as Peak Height Velocity (PHV) to better distinguish training-induced adaptations from natural growth-related processes and to more precisely contextualize developmental timing [54,55]. Finally, additional mechanistic measures, such as force–time characteristics, muscle–tendon imaging, or neuromuscular activation assessments may help explain the proportion of adaptation not captured by contact-time mediation and further clarify predictors of individual responsiveness [56].

## 5. Conclusions

This longitudinal analysis demonstrates that elite youth badminton players can achieve substantial and predominantly linear improvements in explosive strength, reactive strength, and multidirectional speed when exposed to a structured, long-term training process. Despite persistent baseline performance advantages in older athletes, rates of adaptation were largely equivalent across pre-adolescent and adolescent groups, supporting a parallel developmental trajectory rather than a puberty-driven catch-up effect.

Mechanistically, training-induced improvements were characterized by enhanced stretch-shortening cycle function, reflected in increased reactive indices and musculotendinous stiffness. Importantly, gains in jump performance were driven primarily by increased force-production capacity rather than large reductions in ground contact time, highlighting the central role of neuromuscular force expression in long-term athletic development. Substantial inter-individual variability in training responsiveness was evident and could not be explained by chronological age alone, underscoring the importance of continuous monitoring and individualized training adjustments.

Together, these findings emphasize that long-term, well-progressed training can support sustained neuromuscular development across youth badminton pathways and that individualized, mechanism-informed monitoring may be more informative than age-based expectations when optimizing performance development in elite young athletes. These findings further highlight the importance of sensor-based monitoring systems in capturing longitudinal adaptations in neuromuscular performance.

## Figures and Tables

**Table 1:** Pearson Correlation Matrix—Performance Variables and Anthropometric Measures.

Variables	r	95% CI	*p*-Value	r^2^	N
Within Neuromuscular Domain					
CMJ↔Contact Time	−0.687	[−0.768, −0.584]	<0.001 ***	0.472	150
CMJ↔Reactivity	0.534	[0.396, 0.652]	<0.001 ***	0.285	150
Reactivity↔Stiffness	0.512	[0.370, 0.633]	<0.001 ***	0.262	150
Avg Rebound↔CMJ	0.623	[0.495, 0.726]	<0.001 ***	0.388	150
Cross-Domain (Speed-Power)					
Linear 5 m sprint Speed↔CMJ	−0.643	[−0.731, −0.534]	<0.001 ***	0.413	150
Slide Step Right↔Reactivity	−0.418	[−0.555, −0.257]	<0.001 ***	0.175	150
Backward Speed↔Contact Time	−0.456	[−0.587, −0.302]	<0.001 ***	0.208	150
Anthropometric Relationships					
CMJ↔Body Mass	0.458	[0.296, 0.595]	<0.001 ***	0.210	150
Left Leg Jump↔BMI	0.382	[0.211, 0.529]	<0.001 ***	0.146	150
Stiffness↔Body Mass	0.267	[0.079, 0.434]	0.006 **	0.071	150
Directional Movement Coherence					
Linear 5 m sprint Speed↔Backward Speed	0.734	[0.637, 0.812]	<0.001 ***	0.539	150
Slide Right↔Slide Left	0.687	[0.579, 0.774]	<0.001 ***	0.472	150
Cross Right↔Cross Left	0.654	[0.539, 0.747]	<0.001 ***	0.428	150
Weak/Non-Significant Relationships					
Backward Speed↔Stiffness	0.156	[−0.025, 0.322]	0.089	0.024	150
Slide Step↔CMJ	0.234	[0.046, 0.410]	0.016	0.055	150

r—Pearson correlation coefficient; *p*—two-tailed significance level; N—number of observations (150 represents pooled observations across five measurement time points 30 athletes × 5 time points). Confidence intervals (95% CI) are reported for correlation coefficients. r^2^—indicates the proportion of shared variance. ** *p* < 0.01; *** *p* < 0.001 (Bonferroni-corrected α = 0.001).

**Table 2:** Values are presented as n/N (%). χ^2^ = chi-square statistic from Pearson’s chi-square test; *p* = two-tailed significance level. Responders were defined as athletes demonstrating an improvement ≥ 1 SD from baseline in the respective performance variable. Multi-domain responders were classified as athletes meeting responder criteria in ≥2 performance domains (CMJ height, Linear 5 m sprint speed, Reactivity). Broad responders were defined as athletes showing improvement ≥ 1 SD across all three domains. No statistically significant age-group differences were observed for any responder classification (all *p* > 0.05).

Classification Criterion	Older Group	Younger Group	Overall	χ^2^	*p*
CMJ Improvement ≥ 6.0 cm	13/15 (86.7%)	14/15 (93.3%)	27/30 (90.0%)	0.67	0.412
Linear 5 m sprint Speed Improvement ≥ 0.12 s	12/15 (80.0%)	12/15 (80.0%)	24/30 (80.0%)	0.00	1.000
Reactivity Improvement ≥ 0.53	11/15 (73.3%)	13/15 (86.7%)	24/30 (80.0%)	1.23	0.267
Multi-domain Responders (≥2 domains)	10/15 (66.7%)	12/15 (80.0%)	22/30 (73.3%)	1.23	0.267
Broad Responders (all 3 domains)	7/15 (46.7%)	9/15 (60.0%)	16/30 (53.3%)	0.89	0.346

χ^2^—Pearson’s chi-square test statistic used to examine differences in responder proportions between age groups; *p*—two-tailed probability value indicating the likelihood that observed group differences occurred by chance.

**Table 3:** Mediation analysis of training-induced changes in countermovement jump (CMJ) height with contact time as a mechanistic mediator.

Pathway	Coefficient	SE	t/z	*p*	95% CI
Total Effect (c-path-Time → CMJ)					
Time → CMJ	1.500	0.089	16.87	<0.001	[1.324, 1.676]
Indirect Pathway (a-path-Time → Contact Time)					
Time → Contact Time	−2.0	0.254	−7.87	<0.001	[−2.51, −1.49]
Mediator Effect (b-path-Contact Time → CMJ)					
Contact Time → CMJ	−0.345	0.067	−5.15	<0.001	[−0.479, −0.211]
Indirect Effect (a × b product)					
Indirect effect	0.690	0.157	4.39	<0.001	[0.382, 1.004] ^b^
Direct Effect (c′-path-Time → CMJ, adjusted)					
Direct effect	1.810	0.142	12.75	<0.001	[1.912, 2.468]
Total Effect Decomposition					
Proportion Mediated				31.5%	[17.4%, 45.8%]
Proportion Not Mediated				68.5%	[54.2%, 82.6%]

SE = standard error; CI = confidence interval; *p* = two-tailed significance level. Indirect effects were estimated using bootstrap resampling (5000 samples).

## Data Availability

The original contributions presented in this study are included in the article. Further inquiries can be directed to the corresponding authors.

## Abbreviations

The following abbreviations are used in this manuscript:

YG	Younger Group
OG	Older Group
CMJ	Countermovement Jumps
SLJ	Single Leg Jumps
SSC	Stretch-shortening Cycle
